# Intestinal endometriosis combined with colorectal cancer: a case series

**DOI:** 10.1186/s13256-017-1537-3

**Published:** 2018-01-30

**Authors:** Masatsugu Ishii, Masashi Yamamoto, Keitaro Tanaka, Mitsuhiro Asakuma, Shinsuke Masubuchi, Hiroki Hamamoto, Hiroshi Akutagawa, Yutaro Egashira, Yoshinobu Hirose, Junji Okuda, Kazuhisa Uchiyama

**Affiliations:** 1 Departments of General and Gastroenterological Surgery, Osaka Medical College, Osaka, Japan; 2 Cancer Center, Osaka Medical College, Osaka, Japan; 3 Department of Pathology, Osaka Medical College, Osaka, Japan

**Keywords:** Intestinal endometriosis, Colorectal cancer, Laparoscopic surgery

## Abstract

**Background:**

Intestinal endometriosis is a common benign disease among menstruating women that affects the intestinal tract.

**Case presentation:**

This case report presents seven Japanese cases of intestinal endometriosis with colorectal cancer treated by laparoscopic surgery. Five of the seven cases reported here are women presenting with bowel obstruction due to colorectal endometriosis with colorectal cancer. It can be confused with serious lesions such as advanced colorectal cancer with peritoneal involvement or invasion of adjacent organs (T4).

**Conclusions:**

Therefore, we should consider the probability that the cause of bowel obstruction is not T4 but intestinal endometriosis. For surgical treatment, we recommend laparoscopic surgery for colorectal resection because of its benefits of differential diagnosis of T4, preserving fertility, and preventing excessive surgical stress. We performed laparoscopic resection in seven patients with intestinal endometriosis and colorectal cancer. These cases demonstrate the difficulty of establishing a differential diagnosis of intestinal endometriosis with colorectal cancer from T4.

## Background

Intestinal endometriosis is histologically defined as the presence of endometrial glands in the intestinal tract [1–4]. This condition may cause dysmenorrhea, chronic pelvic pain, infertility, dyspareunia, and urinary and intestinal disorders during menstrual flow [1–4]. It is a common benign disease among menstruating women. Diagnostic suspicion of intestinal endometriosis is mainly clinical, based on the aforementioned signs and symptoms caused by the disorder. However, the symptoms are rare and the lack of pathognomonic signs makes the diagnosis difficult. Even during intervention we may confuse intestinal endometriosis with T4 colorectal cancer [5]. Complete removal of all lesions should be performed. A laparoscopic surgical procedure is feasible and effective for making a differential diagnosis of colorectal endometriosis from other malignancies, and is associated with improvements of quality of life and renewed fertility. In this report, we present seven cases in which intestinal endometriosis coexists with colorectal cancer.

## Case presentation

We present seven patients with diagnosed intestinal endometriosis and colorectal cancer. There were 25 patients diagnosed with intestinal endometriosis from 1988 to 2010 at the Departments of General and Gastroenterological Surgery, Osaka Medical College. The detailed clinical results for each of the seven Japanese patients are summarized in Table 1. The average age of the patients was 58.8 years (range 51–68 years), and body mass index (BMI) was 21.5 (range 17.2–25.5 kg/m^2^). All patients were interviewed preoperatively about symptoms, abdominal pain, genital bleeding, melena, dyschezia, and fecal occult blood positivity. There were two nulliparae and five multiparae among our cases. Colonoscopy was performed in seven patients, which revealed an ulcerofungating mass in five patients and a polypoid mass in two patients. A previous colonoscopic biopsy did not reveal endometriosis but revealed malignancy except for cases 1 and 4. A computed tomography (CT) scan was also performed in all patients, and showed limited thickening of the wall of the intestine. Case 1 was diagnosed as having gastrointestinal stromal tumor (GIST). For the other patients, intestinal endometriosis was not diagnosed by preoperative examination; however, colorectal cancer was diagnosed. The primary location was the sigmoid colon in two patients, rectosigmoid colon in three patients, ascending colon in one patient, and cecum in one patient. Laparoscopic surgery was performed in all patients. In case 7, the main tumor had severe adhesions with the uterus and at the anal side of this tumor, a hard mass was observed at the serosal side of the rectum. However, the main tumor did not directly invade the uterus, so we performed laparoscopic low anterior resection with only adhesiolysis of the main tumor and uterus. Intraoperative rapid diagnosis revealed that it was intestinal endometriosis, so we could preserve the uterus. The average operative time was 190 minutes and volume of blood loss was 260 ml (Table 2). In case 1, after the Hartmann procedure, a pelvic abscess was recognized by CT image. Case 3 had postoperative surgical site infection. Finally, all surgical resectional specimens indicated endometriosis according to final pathology reports (Table 3). Lung metastasis was present in two patients. Liver metastasis was present in two patients. Lymphovascular involvement by adenocarcinoma was present in five patients. The average number of dissected lymph nodes was 16 (range 7–28). Lymph node metastasis by adenocarcinoma was present in four patients and endometriosis was present in two patients.

**Table 1:** Clinical characteristics of seven patients with intestinal endometriosis and colorectal cancer

	Age (y)	BMI	Parity	ASA	Symptoms and signs	EM history
1	53	17.2	0	1	Genital bleeding	+
2	64	25.5	1	1	Melaena	−
3	51	22.9	1	1	Faecal occult blood	−
4	57	22.4	3	1	Faecal occult blood	+
5	68	20.7	0	1	Melaena, abdominal pain	−
6	61	20.0	2	1	Faecal occult blood	−
7	58	22.4	1	1	Melaena, abdominal pain	−

*BMI* body mass index, *EM* endometriosis, *ASA* American Society of Anesthesiologists

**Table 2:** Intraoperative and postoperative data after laparoscopic and laparotomic resection of the endometriosis

Procedure	Time (min)	Bleeding (ml)	Blood transfusion	Complication	Complication	Recurrence
Hartmann	285	1725	+	+	Pelvic abscess	+
Lap-AR	170	10	None	None	None	None
Lap-LAR	185	10	None	+	SSI	None
Lap-SD	145	10	None	None	None	None
Lap-RHC	165	10	None	None	None	None
Lap-RHC	135	10	None	None	None	None
Lap-LAR	245	50	None	None	None	None

*Lap* laparoscopic, *AR* anterior resection, *LAR* low anterior resection, *SD* sigmoidectomy, *RHC* right hemicolectomy, *SSI* superficial incisional surgical site infection

**Table 3:** Clinical data of endometriosis in seven patients with colorectal cancer based on the TNM classification of the Japanese General Rules

Location	Diagnosis	Depth	Type	Size (mm)	N	M	H	ly	v
S	GIST	SI	2	90	1	1(LM)	0	1	1
Rs	RK	SM	0–IIa	15	0	0	0	0	0
Rs	RK	SS	2	36	2	1(LM)	1	1	2
S	SK	SS	2	33	1	0	1	1	1
A	AK	SM2 (2000 μm)	0–Isp	32	1	0	0	0	1
C	CK	SS	2	36	0	0	0	1	1
Rs	RK	SS	2	48	1	0	0	1	0
Histology		Dissection		Number		Metastasis		Stage	
mod EM		D3		9		1		IV	
wel EM		D2		12		0		I	
wel mod muc EM		D3		20		4		IV	
wel mod EM		D3		7		1		IIIa	
wel mod pap muc EM		D3		28		1		IIIa	
wel mod EM		D3		11		0		II	
wel mod EM		D3		26		3		IIIa	

*S* sigmoid colon, *Rs* rectal sigmoid colon, *A* ascending colon, *C* cecum, *GIST* gastrointestinal stromal tumour, *RK* rectal cancer, *SK* sigmoid colon cancer, *AK* ascending colon cancer, *EM* endometriosis, *SM* submucosa, *SS* sub serosa, *N* lymph node metastasis, *M* distant metastasis, *LM* lung metastasis, *H* liver metastasis, *ly* lymphatic invasion, *v* vascular invasion, *wel* well differentiated adenocarcinoma, *mod* moderately differentiated adenocarcinoma, *muc* mucinous adenocarcinoma

## Discussion

Endometriosis is histologically defined as the presence of endometrial glands and stroma outside the uterus [1–4]. It is a common benign disease among menstruating women [1–4]. The frequency of intestinal endometriosis is estimated to be approximately 3–34% of endometriosis [1–4]. Endometriosis is estimated to affect the intestinal tract in 15–37% of patients with pelvic endometriosis [5–7]. Many theories regarding the pathogenesis of this benign gynecological disease have been proposed. The most widely accepted theory proposes retrograde menstruation and subsequent implantation of regurgitated endometrial cells on the peritoneum and pelvic viscera, which appears to be facilitated by alterations in cell-mediated and humoral immunity [5]. This pathology occurs in women of childbearing age and can be located in various anatomic sites including the peritoneum, ovary, fallopian tubes, cervix, vagina, vulva, rectovaginal septum, uterosacral ligaments, rectosigmoid bowel, bladder, uterus, and skin [5]. Intestinal endometriosis often presents as bowel obstruction due to a submucosal tumor or luminal stenosis because endometrial tissue usually involves the outer walls of the intestinal tract such as the serosal or submucosal layer. In that case, preoperative diagnosis of intestinal endometriosis is very difficult despite the use of CT or magnetic resonance imaging (MRI), and it is important to differentially diagnose intestinal endometriosis from advanced colorectal cancer such as invasion of adjacent organs (T4) [8].

In general, in almost all cases, intestinal endometriosis is diagnosed by histological findings after surgical resection [5]. Actually, in our series, there were no cases of preoperatively diagnosed intestinal endometriosis.

Operation is the basic treatment when treatment is needed for intestinal endometriosis. For surgical treatment of intestinal endometriosis, early detection, resection of endometrial tissue, and relief of symptoms are important. Although lymph node involvement by endometriosis is considered to be uncommon, there are a few reports of endometrial cells in the lymph nodes, despite intestinal endometriosis itself not being considered a malignant disease [9]. Therefore, lymph node dissection is required as well as resection of primary endometrial tissue.

In cases of T4 malignant tumor, intestinal resection with a safety margin and dissection along with a salpingo-oophorectomy and hysterectomy may be necessary [10]. However, in cases of intestinal endometriosis, which is a benign disease in reproductive women with the full thickness of the intestinal wall with no evidence of malignancy, preserving the uterus and ovary is very important and a laparoscopic approach has great potential as a diagnostic tool [10].

In recent years, laparoscopic surgery for colorectal disease has been appreciated and this technique has been improved; the procedure is also effective for distinguishing intestinal endometriosis from colorectal cancer [11]. Because of the ability of the laparoscope to zoom in and out in the pelvic space, it is possible to diagnose intestinal endometriosis during surgery. Therefore, laparoscopic surgery has great potential to contribute to preserving fertility and reducing invasiveness.

Several studies have shown that laparoscopic colorectal resection for endometriosis is feasible and safe, including a randomized prospective study by Darai and colleagues [12]. We should consider the patient’s age and fertility, and complications of the disease. Pelvic endometriosis sometimes involves the intestinal tract and a malignant tumor. When an unexpected pelvic mass lesion is found during surgery, an intraoperative histological examination should be considered for conclusive diagnosis.

In all of our cases, patients were not diagnosed preoperatively. In case 1, preoperative diagnosis was GIST, and it caused severe stenosis of the intestinal tract. In the other cases, preoperative diagnoses were colon cancer; however, they were diagnosed with intestinal endometriosis histologically. In case 7, we were able to preserve the uterus as rapid diagnosis and laparoscopic observation revealed that there was no invasion by T4 tumor. Furthermore, a previous study described that early diagnosis by intensive endoscopic examination could improve the number of curative colorectal resections [13]. In addition, the use of intraoperative colonoscopy is also very useful in that an adequate cutting line can be obtained by checking mucosal findings.

In our group, we performed laparoscopic surgery for 1433 colorectal cancer cases from November 2003 to June 2010. We consider laparoscopic surgery to be valid for observation of the affected part and pelvic space; therefore, it represents a safe approach for the treatment of intestinal endometriosis or colorectal cancer. Our postoperative morbidity and mortality rate is in accordance with many recent reports [14–17]. Our experience will be related to a suitable intraoperative decision for preserving fertility and preventing excessive surgical stress.

We have performed laparoscopic surgery in combination with intraoperative endoscopy, which discloses the safety margin and other masses of the intestine. Especially in the case of laparoscopic low or super-low anterior resection, the edge of the incision line could be determined correctly using this technique in combination with intraoperative endoscopy.

## Conclusions

In conclusion, intestinal endometriosis with colorectal cancer is a relatively rare disease and is difficult to distinguish from T4 cancer. Therefore, we should consider the probability that the cause of bowel obstruction is not T4 but intestinal endometriosis. For surgical treatment, laparoscopic resection of the rectum or colon for intestinal endometriosis can be performed safely and effectively. Therefore, we consider that our laparoscopic approach with intraoperative endoscopy as a standardized technique for this disease will be increasingly indicated.

## Abbreviations

BMI: Body mass index
CT: Computed tomography
GIST: Gastrointestinal stromal tumor
MRI: Magnetic resonance imaging
